# The Use of Vedolizumab in Patients with Concomitant Cirrhosis and Crohn’s Disease

**DOI:** 10.7759/cureus.3080

**Published:** 2018-07-31

**Authors:** Nikhil Kapila, Gianina Flocco, Bo Shen, Jamak Modaresi Esfeh

**Affiliations:** 1 Gastroenterology, Cleveland Clinic, Weston, USA; 2 Gastroenterology, Cleveland Clinic, Cleveland, USA; 3 Hepatology, Cleveland Clinic, Cleveland, USA

**Keywords:** inflammatory bowel disease (ibd), crohns disease, liver cirrhosis, biologics

## Abstract

Vedolizumab is a humanized monoclonal α4β7 integrin antibody used in patients with Crohn’s disease (CD) and ulcerative colitis (UC). Limited data are available on the use of vedolizumab in patients with concurrent cirrhosis and inflammatory bowel disease (IBD). Patients with cirrhosis are unique, as they have a predilection for developing opportunistic infections and malignancies. Additionally, it is not known if vedolizumab alters the natural course of cirrhosis. We report our experience in three patients with concomitant CD and cirrhosis, who were treated with vedolizumab. In our limited cohort, all the three patients tolerated vedolizumab well. None of them experienced significant infectious complications, nor did any have decompensated cirrhosis. Our limited series suggest that vedolizumab is well tolerated in patients with compensated cirrhosis.

## Introduction

Vedolizumab is a humanized monoclonal α4β7 integrin antibody that is approved for use in the management of patients with moderate to severe Crohn’s disease (CD) and ulcerative colitis (UC). Long-term safety data on the use of vedolizumab suggest a favorable safety profile with a low incidence of severe infection [1]. However, neither the landmark studies that described the efficacy of vedolizumab nor the recent studies that describe their long-term safety have reviewed the use of vedolizumab in patients with concomitant inflammatory bowel disease (IBD) and cirrhosis [2-3]. Patients with cirrhosis are unique in that they are immunocompromised with an increased susceptibility to infections and have a predilection for hepatic and extra-hepatic malignancies [4]. From January 2014 to September 2018, 1542 patients with IBD were treated with vedolizumab at our center, and three of these patients were identified as having cirrhosis based on liver biopsy, biochemical data, and/or imaging.

## Case presentation

The first patient is a 64-year-old female with a history of CD of the colon and small bowel and cirrhosis secondary to autoimmune hepatitis (AIH). She was diagnosed with CD 13 years earlier and had failed treatment with certolizumab and infliximab, thus requiring a colectomy with end ileostomy. She continued to be symptomatic with significant ileostomy output and arthropathy, and ileoscopic findings were consistent with persistent small bowel CD; therefore, vedolizumab therapy was considered. Prior to starting treatment, she had a modified model for end-stage liver disease (MELD-Na) score of eight and a Child Turcott Pugh (CTP) score of A5. She was started on vedolizumab 300 mg every eight weeks and azathioprine 100 mg daily. Thirty-six months after treatment, she is doing well with mucosal healing on follow-up ileoscopy. The patient has not had any complications related to her cirrhosis and continues to have well-preserved liver function. Her MELD-Na and CTP scores remain unchanged. Surveillance imaging for hepatocellular carcinoma has been negative, and she has not required hospitalization for infectious complications.

Our second case is of a 57-year-old male with a history of small bowel CD and cirrhosis due to chronic hepatitis C infection. Cirrhosis was confirmed by liver biopsy and the patient achieved a sustained virologic response after completing therapy with interferon and ribavirin. He has a five-year history of CD involving the small bowel, had undergone a prior resection of the distal ileum, and has previously been treated with budesonide and 6-mercaptopurine (6-MP). Due to persistent clinical and endoscopic disease in the neoterminal ileum, vedolizumab therapy was considered. Prior to therapy, his MELD-Na score was six and CTP A5. Thirty-two months post treatment, the patient continues on vedolizumab 300 mg every eight weeks and his CD is in clinical remission. He has not had any episodes of hepatic decompensation and his post-treatment MELD-Na score is seven and CTP A6. He has not had any infectious complications, nor has he developed a hepatic or an extra-hepatic malignancy.

The last case is of a 65-year-old male with an 11-year history of small bowel CD and cryptogenic cirrhosis. He was initially treated with steroids, infliximab, and azathioprine; however, due to non-response, vedolizumab therapy was considered. Prior to treatment, his MELD-Na was nine and CTP A6. After initiation of vedolizumab 300 mg every eight weeks for five months, the patient’s MELD-Na was 11 and CTP A6 with no decompensated cirrhosis or significant infection.

## Discussion

Vedolizumab targets the integrin heterodimer α4β7, thus decreasing intestinal inflammation by interfering in intestinal T cell trafficking. By virtue of its intestinal selectivity, long-term data on safety and efficacy suggest that vedolizumab has a favorable safety profile. In a cohort of 294 patients with IBD treated with vedolizumab, five patients developed gastrointestinal infections and one patient was noted to have liver test abnormalities [5]. Another study of 136 IBD patients who were followed for a year showed that 17 patients developed severe infections and no patients had liver test abnormalities [6]. Drugs commonly used in patients with IBD are associated with varying degrees and patterns of liver injury. Azathioprine has been associated with cholestasis and infliximab with hepatocellular injury; however, similar results have not been observed with the use of vedolizumab [7]. Although data on the long-term effectiveness and safety of vedolizumab are increasingly avaliable, its use in patients with concurrent cirrhosis has not been established. Patients with cirrhosis are unique in that their underlying hepatic dysfunction increases their risk of opportunistic infections and both hepatic and extra-hepatic malignancies. Biological agents, such as infliximab, have been associated with an increased frequency of severe infections and death when used in patients with alcoholic hepatitis [8]. In our limited cohort, we did not observe any changes in MELD-Na and CTP scores, nor were there any episodes of hepatic decompensation or significant infectious complications. While other studies have described the efficacy and safety of vedolizumab in patients with concomitant IBD and primary sclerosing cholangitis, our study is the first to describe the use of vedolizumab in patients with cirrhosis and CD [9]. Our experience suggests that vedolizumab is effective and safe in patients with compensated cirrhosis and concurrent CD.

## Conclusions

There is a paucity of data on the use of vedolizumab in patients with concurrent cirrhosis and IBD. These patients are unique, as they have an increased predilection for infectious complications and malignancies. Our experience, however, suggests that vedolizumab is well tolerated and efficacious in patients with concurrent compensated cirrhosis and CD.

## References

[1] Colombel J-F, Sands BE, Rutgeerts P (2016). The safety of vedolizumab for ulcerative colitis and Crohn’s disease. Gut.

[2] Feagan BG, Rutgeerts P, Sands BE (2013). Vedolizumab as induction and maintenance therapy for ulcerative colitis. N Engl J Med.

[3] Sandborn WJ, Feagan BG, Rutgeerts P (2013). Vedolizumab as induction and maintenance therapy for Crohn’s disease. N Engl J Med.

[4] Bonnel AR, Bunchorntavakul C, Reddy KR (2011). Immune dysfunction and infections in patients with cirrhosis. Clin Gastroenterol Hepatol.

[5] Amiot A, Grimaud J-C, Peyrin-Biroulet L (2016). Effectiveness and safety of vedolizumab Induction therapy for patients with inflammatory bowel disease. Clin Gastroenterol Hepatol.

[6] Christensen B, Colman RJ, Micic D (2018). Vedolizumab as Induction and Maintenance for Inflammatory Bowel Disease: 12-month Effectiveness and Safety. Inflamm Bowel Dis.

[7] Koller T, Galambosova M, Filakovska S (2017). Drug-induced liver injury in inflammatory bowel disease: 1-year prospective observational study. World J Gastroenterol.

[8] Naveau S, Chollet-Martin S, Dharancy S (2004). A double-blind randomized controlled trial of infliximab associated with prednisolone in acute alcoholic hepatitis. Hepatology.

[9] Christensen B, Micic D, Gibson PR (2018). Vedolizumab in patients with concurrent primary sclerosing cholangitis and inflammatory bowel disease does not improve liver biochemistry but is safe and effective for the liver disease. Aliment Pharmacol Ther.

